# Understanding knowledge and media influence on people with hepatitis B in Senegal: a mixed-methods study

**DOI:** 10.1136/bmjopen-2024-085453

**Published:** 2025-03-24

**Authors:** Albert Gautier Ndione, Mariama Diédhiou, Séverine Carillon, Judicaël Malick Tine, Amady Ndiaye, Louise Fortes, Ndèye Fatou Ngom, Moussa Seydi, Gilles Wandeler, Adrià Ramírez Mena, SEN-B investigators, Amady Ndiaye

**Affiliations:** 1 Sociology Department, Cheikh Anta Diop University of Dakar, Dakar, Senegal; 2 Centre Régional de Recherche et de Formation à la prise en charge clinique de Fann, Fann University Hospital, Dakar, Senegal; 3 Service des Maladies Infectieuses et Tropicales, University Hospital Fann, Dakar, Senegal; 4 Department of Infectious Diseases, Dalal Jamm Hospital, Guediawaye, Senegal; 5 Département de Médecine, UFRSDD de l'UAD de Bambey, Centre de Traitement Ambulatoire, University Hospital Fann, Dakar, Senegal; 6 Department of Infectious Diseases, Bern University Hospital, University of Bern, Bern, Switzerland; 7 Institute of Social and Preventive Medicine, University of Bern, Bern, Switzerland; 8 Department of Infectious Diseases, Inselspital University Hospital Bern, Bern, Switzerland; 9 Graduate School of Health Sciences, University of Bern, Bern, Switzerland

**Keywords:** Chronic Disease, Internet, Social Media, SOCIAL MEDICINE

## Abstract

**Abstract:**

**Objectives:**

Public awareness and the dissemination of tailored information to lay populations are essential for highly endemic countries like Senegal to achieve hepatitis B elimination targets by 2030. In Senegal, despite its high prevalence, hepatitis B has not received sufficient attention in health communication campaigns compared with other health issues like HIV. We aimed to explore knowledge and perceptions surrounding hepatitis B virus (HBV), as well as the influence of digital media on the information accessed by individuals living with HBV in Senegal.

**Design:**

We employed a mixed-methods approach combining qualitative semistructured interviews conducted with people living with HBV enrolled in the Senegalese hepatitis B cohort (SEN-B), with a quantitative content analysis of online news coverage focused on HBV within the online media of Senegal.

**Setting:**

A referral University hospital in Dakar, Senegal.

**Participants:**

29 individuals aged >18 years presenting with a positive hepatitis B surface antigen (HBsAg) with a median age of 40 years (IQR 27–54), of whom 51.7% were female.

**Outcomes and analysis:**

Qualitative interviews were conducted between December 2019 and October 2021, and we employed purposive sampling to select participants enrolled in SEN-B. Thematic analysis facilitated a systematic synthesis of respondents’ narratives. All data analyses were performed using Atlas.ti (V.22). For content analysis of online media news collected from September 2019 to May 2022, a structured data extraction form was developed to collect relevant information from the selected online news articles. Data on readers’ comments spaces were extracted using an inductive approach and were processed using thematic analyses. The quantitative data issued from content analysis were exported to Stata SE V.17.0 (StataCorp) for statistical analysis.

**Results:**

We observed a generalised lack of knowledge about HBV among participants, some of whom had never heard of the virus prior to their screening. Incomprehension regarding the disease contributed to feelings of fear and anxiety, leading participants to express various concerns about their personal health status, transmission, cure and treatment(s). The presence of rumours surrounding the disease further underscored the limited awareness of HBV revealing the marginal recognition of HBV as a significant societal concern. In many cases, the absence of effective health communication strategies at the national level resulted in individuals turning to traditional and online media for information, which often intensified their fears and concerns about HBV. An analysis of Senegalese media coverage about HBV included 157 articles published between 2009 and 2022. 55.4% (87/157) of these publications appeared in July, coinciding with World Hepatitis Day, while 65.0% (102/157) focused on general HBV epidemiology and activities led by the National Hepatitis Programme. Online media also served as informal spaces where unaccredited actors within the health sector promoted treatments lacking official verification. Additionally, the reactions’ spaces provided a venue for the exchange of information, though without any guarantee of its accuracy.

**Conclusions:**

Facilitating collaboration and engagement between health communication stakeholders and communities is crucial for effectively disseminating structured information and culturally appropriate messages, ultimately contributing to raising awareness of HBV.

**STRENGTHS AND LIMITATIONS OF THIS STUDY**

- We adopted a mixed-methods approach that integrated qualitative interviews with individuals at various levels of hepatitis B virus (HBV) care alongside media content analysis, all ensuring a comprehensive understanding of the knowledge and perceptions surrounding hepatitis B in Senegal.
- The study did not investigate the perspectives of other significant stakeholders in health communication, including healthcare professionals and online media journalists.
- Participants interviewed have been engaged in care prior to the Senegalese hepatitis B cohort cohort enrolment, and their experiences may not fully represent either those of the population living with HBV or those of the general population.
- The content analysis focused on primary themes but did not thoroughly explore the entire range of topics present in the analysed online media articles.

## Introduction

 Chronic hepatitis B virus (HBV) infection continues to represent a significant public health burden in West Africa.^1^ In Senegal, HBV affects more than 10% of the general population, contributing to an elevated risk of mortality from cirrhosis and liver cancer.^2 3^ Despite the availability of vaccination and effective antiviral therapy, the majority of individuals remain unaware of their HBV infection status, thus limiting early detection, prevention and access to timely care and treatment.^4^ In 2016, the WHO set ambitious goals for the elimination of viral hepatitis, including HBV, as a public health threat by 2030. However, achieving these objectives in the WHO AFRO region presents unique challenges due to the lack of effective strategies to communicate about HBV and insufficient resources for healthcare.^5^ Although standardised blood testing protocols exist, current practices failed to identify persons with chronic HBV infection and to ensure comprehensive counselling and linkage to care, revealing a systemic gap in the implementation of WHO screening policies and patient-centred healthcare practices.^6 7^

In recent years, the rapid expansion of digital media has facilitated greater access to health information in sub-Saharan Africa, with Senegal demonstrating high rates of internet penetration.^8^ The introduction of the internet in the mid-1990s transformed the Senegalese landscape of health communication, facilitating the emergence of online platforms focusing exclusively on health-related information. Press organisations’ websites, which we refer to as ‘online media’, adopted various modalities, including intermediaries (web aggregators), traditional print media adapted for the web (companion websites) and websites that originated online (pure players). Furthermore, healthcare stakeholders including medical professionals, traditional healers, entertainers and associations involved in health and media communication were instrumental in the evolution of these online media.^9 10^ However, the expansion of online information sources has led to challenges in effectively prioritising and organising information pertaining to specific health conditions, such as hepatitis B.^11^ Throughout the recent SARS-CoV-2 pandemic, digital media proved to be a valuable tool for disseminating health information and raising public health awareness.^12 13^ Although they have brought about significant advancements, their expansion has also given rise to concerns and setbacks including misinformation, rumours and ‘infodemics’, and the promotion of unproven treatments.^14^,^16^

Health communication strategies, as part of national hepatitis control programmes policies, have recurrently been under-resourced in Western Africa, which has prevented widespread awareness and understanding of HBV among the lay population.^5^ Moreover, insufficient knowledge among healthcare practitioners, the confusion with other diseases and the attribution of symptoms to occult forces are among factors that challenge appropriate HBV knowledge.^17^,^19^ In Senegal, while the management of other public health threats such as HIV and malaria has historically benefited from robust national policies, formal information and regulatory structures, the same level of support is lacking for HBV.^18 20^ Within the urgent context of hepatitis B elimination by 2030, the fundamental preliminary stage—even prior to screening and treatment—namely, access to information, exhibits critical gaps in Senegal which require immediate attention. This study consequently contributes to delineating the current landscape of hepatitis B information, establishing essential groundwork for its restructuring within national awareness programmes.

### Objectives

In the present study, we aimed to investigate the extent of awareness and knowledge regarding HBV by examining the experiences and perspectives of individuals living with HBV in Senegal. Furthermore, we sought to analyse the role of online media in disseminating health information and knowledge about HBV.

## Methods

### Study design

This study is a mixed-methods study with an embedded design (EMM), which combines the collection and analysis of both quantitative and qualitative data, where one method is prioritised, and the other one is used to supplement it (online supplemental figure 1).^21^ We combined interviews with people living with HBV and a content analysis of online media, where data were collected and analysed concurrently. This integration of the different sources of data occurred during the interpretation phase.

### Study site and participants

We collected qualitative data through semistructured interviews with a sample of individuals enrolled in the Senegalese hepatitis B cohort (SEN-B), a prospective cohort study of adult individuals living with HBV followed at the Infectious and Tropical Diseases Service at Fann University Hospital in Dakar, Senegal.^2^ Participants are followed biannually to assess their eligibility for antiviral treatment, as well as for clinical monitoring and hepatocellular carcinoma surveillance using abdominal ultrasound. At the initial visit, all SEN-B received information and counselling on a wide range of HBV-related topics, including transmission risks, antiviral treatment, disease progression, prevention strategies and family screening strategies were encouraged.

All participants in this present study agreed to participate in our qualitative investigation on a voluntary basis, and no clinical intervention was associated with this study.

### Qualitative interviews and data analysis

We included 29 SEN-B participants for semistructured interviews conducted between 2019 and 2021. The 2-year period of qualitative data collection was impacted by the COVID-19 pandemic lockdown in Senegal, which disrupted clinical and research activities. We employed purposive sampling to select participants based on specific criteria including sex, age, education and the length of time since they became aware of their HBV positive status and their inclusion in SEN-B. An exploratory phase included interviews with nine individuals to develop and test the interview guide. The interviews covered topics such as HBV screening practices, disease announcement, disclosure patterns, information and knowledge of HBV, access to care and disease perceptions. These insights were used to refine the interview guide for the present study (online supplemental file 1). The second phase, conducted in 2021, included 20 individuals using the validated guide. The interviews began with the following initial questions: “When you hear the term “hepatitis B” what thoughts come to mind?” and “What questions do you have regarding hepatitis B?”. The average duration of the interviews was 1 hour and 10 min, until information saturation was reached. To accommodate the language preferences of the participants, interviews were conducted in either French or Wolof. All interview sessions were recorded to capture the entire conversation accurately. The recorded interviews were then transcribed verbatim into French by a bilingual expert, ensuring an exact representation of the spoken content. Coding was conducted using both deductive and inductive approaches. Thematic analysis allowed for a structured synthesis of the respondents’ narratives. Selected narratives included in this article were translated from French to English, with quality assurance checks conducted, and the translated narratives compared with the original French versions. All data analyses were performed using Atlas.ti (V.22).

### Content analysis of online media

We conducted a quantitative content analysis to investigate the representation of hepatitis B in Senegalese online media to further explore the relationship between HBV media coverage and public understanding of the disease. We synthesised data from Senegalese media websites specifically focusing on the coverage of hepatitis B.^22^ To ensure systematic data collection, a structured data extraction form was developed to collect relevant information from the selected online news articles (online supplemental file 2). We used a ‘forward-backwards’ search strategy to select articles. The first step (‘forward’) consisted in retrieving articles using an advanced Google search strategy including the following keywords (in French): “Hépatite” OR “Hépatite B” AND “Sénégal”. Articles were selected for the analysis based on the following inclusion criteria: (1) relevance to the subject of hepatitis B, (2) focus on Senegal, (3) published in a Senegalese digital media source and (4) published in French. We excluded all articles published in other web platforms such as personal blogs, academic, institutional or (trans)national agencies’ websites or other websites not identified as digital media. Furthermore, we monitored all newly published articles using a predefined ‘Google search alarm’ from September 2019 to May 2022. In a subsequent phase (‘backwards’), we explored additional articles by introducing the selected keywords in the “Search boxes” of all online media websites previously identified. Two researchers (AGN and ARM) independently assessed the individual articles for eligibility, quality and validity and captured data on the sources, publication dates, content themes and the presence of accurate information related to HBV (online supplemental file 3). The content themes were extracted by one researcher (AGN) using an inductive approach and were cross-checked by a second researcher (ARM) to ensure consistency. Data on readers’ comments spaces were extracted using an inductive approach and processed using thematic analyses. All qualitative analyses were conducted using Atlas.Ti (V.22). The quantitative data issued from content analysis were exported to Stata SE V.17.0 (StataCorp) for statistical analysis. Descriptive statistics such as frequencies (n), proportions (%) and numerical summary measures (including means and SDs) were used to describe the data.

### Data integration

The integration of both qualitative and quantitative findings was conducted through a process of triangulation, which involved comparing and contrasting the results from the different data sources to provide insights into the alignment or discrepancies between individual perspectives and media representations of HBV in Senegal.

### Patient and public involvement

The patient association Saafara Hépatites Sénégal (SH), which represents individuals with hepatitis B in Senegal, played a key role in developing and implementing the study protocol. Members of SH serve as peer counsellors, provide support to participants and hold formal propositions on SEN-B’s scientific board, thereby influencing strategic decisions. The study results will be shared at conferences and with participants.

## Results

### Study population

The sociodemographic characteristics of the 29 persons interviewed are presented in table 1. Their median age was 40 years (IQR 27–54), 15 (52%) were female, 19 (67%) were married, 12 (42%) had less than secondary education and 18 (62%) were aware of their HBV status more than 2 years prior to the interview.

**Table 1:** Characteristics of interviewed participants

Characteristics	n=29	(%)
Sex		
Male	14	48
Female	15	52
Age (years)		
18–30	10	35
31–50	6	21
>50	13	45
Marital status		
Single	8	28
Married	19	65
Divorced	1	4
Widowed	1	4
Educational level		
None	4	14
Primary	8	28
Secondary	5	17
Tertiary	11	38
Employment		
None	3	10
Student	6	21
Formal	6	21
Informal	14	48
Time since HBV diagnosis (years)		
0.0–2.0	11	38
2.0–5.0	8	28
>5.0	10	35
On HBV antiviral therapy		
No	26	93
Yes	2	7

HBVhepatitis B virus

Three major themes emerged from the interviews: (1) the absence of specific cultural representations of HBV, primarily due to a lack of structured and formal information sources, (2) the anxieties and apprehensions surrounding the disease, derived from fear of the unknown, circulating rumours and misconceptions and (3) the utilisation of various information sources, including the internet, as a means of accessing health information.

### Lack of distinct representations of HBV

The participants’ collective representations revealed an absence of identifiable cultural or symbolic connotations surrounding HBV. This lack of association was primarily due to their unfamiliarity with HBV within their specific cultural context. The circulating rumours, coupled with the absence or limited availability of official information from health-related institutions, resulted in a lack of widely shared knowledge, which could have facilitated representations of HBV.

I don't have much knowledge about the disease, and that’s why it scares me. (Female, early 50s, married)

I was devastated because I didn't know what hepatitis B was. I'd never heard of it, I'd never seen anyone in my family who had it. I never paid any attention to it. (Female, early 40s, married)

Participant narratives and lived experiences highlighted widespread acknowledgement of the neglect of HBV as a public health concern among the general Senegalese population.

It’s a disease that’s not well known everywhere in Senegal, but the Senegalese underestimate it. They don't know about this virus. (Male, late 20s, married)

### Concerns expressed regarding hepatitis B

Amidst prevailing unawareness, individuals find themselves immersed in a sphere of fear and uncertainty, prompting them to raise numerous concerns. A strong desire for knowledge was reported by most participants, as they had been unsuccessfully seeking answers and information following their diagnosis. Participants’ inquiries about hepatitis B primarily focused on three key aspects: the viral infection’s progression within their body, the risk of transmission to their close relatives and the prospects of disease recovery. They sought comprehensive information about the disease’s clinical trajectory and its potential long-term complications. Nutritional management emerged as a significant area of interest, with participants requesting detailed guidance on dietary strategies that might influence the disease’s progression and management.

My priority is really to find out if it’s curable [hepatitis B], that’s the most important thing. I need to be told that the disease has been eradicated from my body, or that the virus is this way or that way, so that I know for sure. So far, I have many doubts. (Male, early 20s, single)

The pursuit of knowledge was often perceived as essential, yet it coexisted with apprehension, resulting from the anticipation of distressing information.

Tell me, is this disease contagious? I really want to know. (…) I want to know if it is possible to cure or not, that’s all I’m interested in. But I am afraid because I don't want to be told something that will scare me even more. (Female, early 50s, married)

External sources of information, such as rumours, frequently disseminate incomplete and unreliable medical messages regarding critical aspects of HBV management, particularly concerning treatment options. These constructed narratives lead to significant (mis)interpretations among individuals, highlighting a gap in effective communication from healthcare practitioners.

I've heard people say that when you start taking medication, it’s lifelong, but I haven't discussed this with doctors or anyone else; and what you do take for life is for something that can't be cured. It seems that when an illness becomes chronic, it can't be cured. (Female, late 30s, divorced)

Religious beliefs were mentioned by the majority of the participants as coping mechanisms to confront the anxieties arising from a lack of information.

I would like us to be well informed about the cure options and the transmission of the disease. I want the doctors to talk about it, to tell us what to do. (…) I'm not afraid of anything, and I rely on God’s will for everything that will happen to me. (Female, late 30s, divorced)

Concerns about stigmatisation, based on the visible physical signs of the disease, were also mentioned. It was noted that, in some cases, the fear of stigmatisation was even greater coming from people who would certainly not help and would only gossip on one’s situation.

Will the disease hinder me from living a normal life and be evident on my body, because people will judge me and then they will not help me? (Female, early 50s, Widow)

Women and men had different concerns about HBV transmission. Among women, there was a distinct preoccupation with the transmission of the disease while breastfeeding their infants. In contrast, men exhibited concerns over potential transmission through their own blood. This particular belief came from the conviction that a child could ‘inherit the same blood’ as his father, conceptualising HBV transmission as a hereditary process.

How can someone marry a person without transmitting HBV to their spouse and then have children with that person without transmitting the disease to the children? (Male, late 20s, single)

Younger individuals raised concerns about the impact of HBV on their physical activities. Hepatitis B infection was also perceived as a constraint to engaging in professional or recreational activities that could require physical effort.

I have submitted [an application] for recruitment to the gendarmerie. Currently, I am preparing for the competitive examination… Will my illness pose any obstacles during the process? If I successfully pass the exam, will I be able to continue my treatment? (Female, mid 20s, single)

### Sources of information and the ripple effect of fear

Generally, participants sought information from the very moment they received their test result. Hepatitis B screening was predominantly incidental, with most cases discovered during routine activities such as blood donations, general healthcare interactions or prenatal examinations. Often, individuals were not specifically seeking testing in the context of hepatitis-related symptoms. For others, the lack of pretest counselling and specific information on HBV after the testing was perceived as a synonym of ‘ordinary illness’, and the process of searching for information often only started after a close relative’s death was attributed to HBV.

The first person to die was a half-brother on my mother’s father’s side. When the second person died, they said it was from the same disease. That terrified me. I started searching for information online, fearful of what I might discover. (Female, mid 20s, single)

I never heard about hepatitis before the announcement (…). It was after the announcement that I started paying attention. One day, (…) I watched a TV program about hepatitis B. The journalist said she knew people who had died of hepatitis (…) They say a lot of things and it’s confusing. (Female, Female, mid 20s, single 50s, married)

Traditional mass media, including television and radio, were frequently mentioned as a primary source of information. However, the information received was perceived as vague and not always reliable. The dissemination of distressing messages through these media was often perceived as a catalyst of fear and apprehension.

It was a radio broadcast. I don't remember if it was on RFM or Radio Sénégal. …You know, they don't really help to appease people in this sense: they say it can kill you when your liver blackens, when you are not aware of it yet. (Female, mid 20s, married)

I watched a TV report about a woman who had the disease without knowing it. She finally got liver cancer and died. All these things scare me and I don't want to think about it. (Female, late 20s, married)

The absence of precise information at the time of the announcement of the diagnosis commonly triggered feelings of fear and confusion among individuals, leading them to seek new information from alternative sources or engage in internet searches.

They didn't tell me anything about [hepatitis B] at the national blood bank. When they told me that I could no longer donate, at that moment I said to myself that there was something wrong. I cried so much because I had no information about the disease. I was scared. I told myself that it was better to look for information. I started to do my own research on Google. (Female, mid 30s, married)

After the doctor told me I had hepatitis B, I went to a friend’s house. I spent the whole day looking on the internet. I watched a video on Youtube that explained what hepatitis B is, how it manifests itself, and how you contract it… I looked at everything. (Female, early 40s, married)

While using ‘Google’' as a search engine, participants often came across news articles, videos and scientific documents that did not consistently provide reliable and clear information. In some instances, the information contradicted established biomedical knowledge, resulting in a growing distrust towards healthcare institutions and practitioners.

I heard information about hepatitis on the radio. What I heard on the radio is different from what is here [clinic]. I heard (…) that patients will be on treatment for three months, but I have not seen that here. (Male, late 50s, married)

HBV-related information was also conveyed through informal sources, particularly rumours and gossip. The dissemination of personal interpretations and knowledge about the disease often led to the circulation of conflicting statements within the community.

People on the street no longer want to share the same drinks because there are so many cases of hepatitis. They think it’s transmitted through saliva. But there’s no proof of that, I've asked the doctors. Out there, there’s a street language. Everyone talks about hepatitis, but they don't have the right information. (Male, early 30s, single)

Rumours, regardless of their accuracy, pushed individuals to adopt strict practices and behaviours that were often not aligned with biomedical recommendations.

### Hepatitis B coverage in online media: content analysis

**Figure 1:**
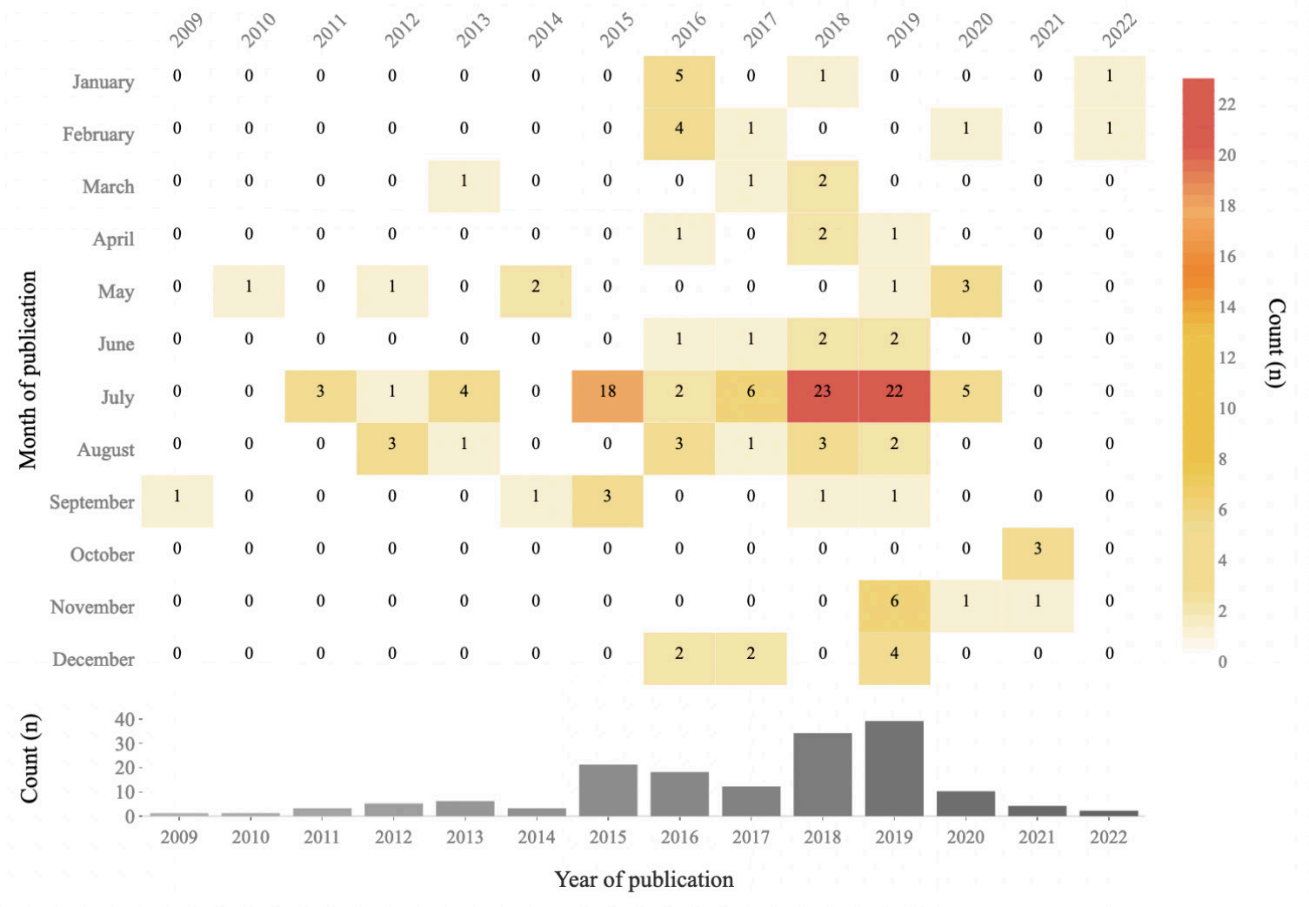
Number of HBV-related articles extracted from Senegalese digital media over time, by month and year of publication. HBV, hepatitis B virus.

We analysed 157 articles issued from the content analysis of Senegalese news media published online between 2009 and 2022. The total number of recorded online media websites was 62 with an average number of articles per website of 2.57 (± SD 2.67) (online supplemental file 3). The number of readerships was available for 18 articles and comments or replies for 17 articles, accounting for 180 330 views and 214 comments. The distribution of the number of eligible articles over time showed that the number of yearly publications increased between 2009 (1 article) and 2019 (39 articles) and decreased after the beginning of the SARS-CoV-2 pandemic (10 in 2020, 4 in 2021 and 2 in 2022) (figure 1). Overall, 84 (53.5%) articles were published in July, corresponding to the period of the celebration of World Hepatitis Day (28 July).

**Figure 2:**
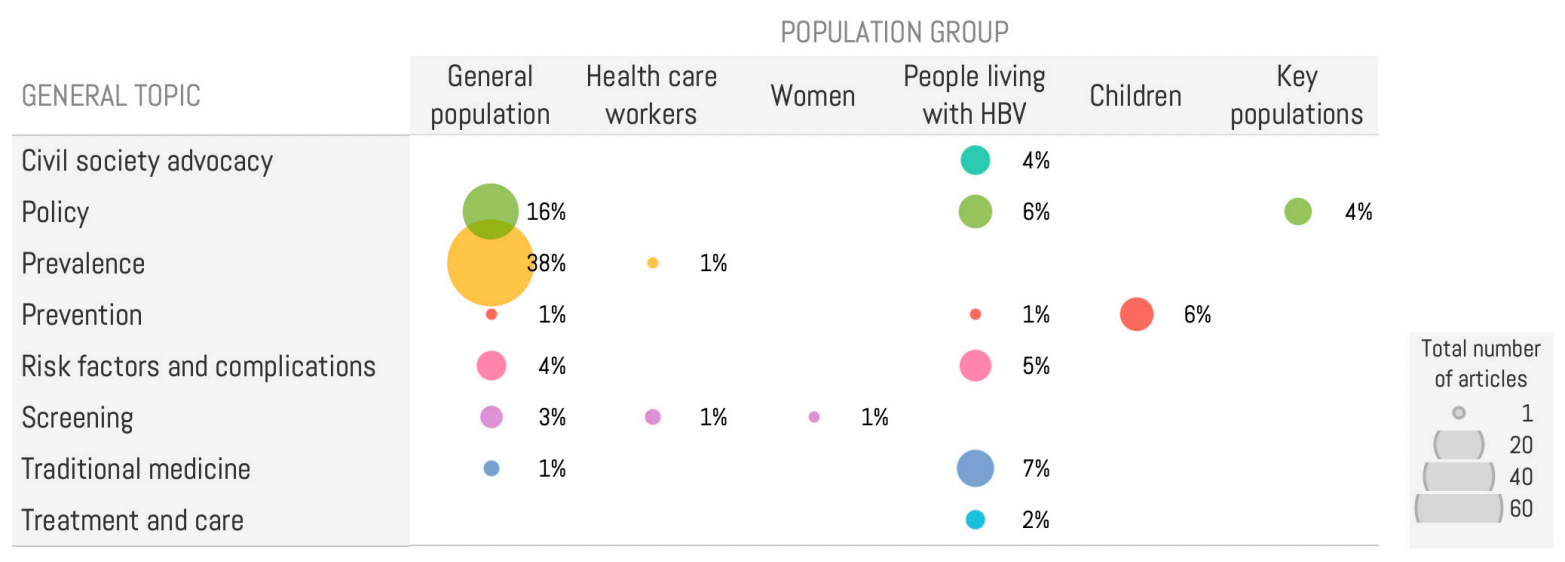
Distribution of general topics issued from content analysis of Senegalese digital media, categorised by the referenced population groups.

Eight general topics related to HBV emerged from our analysis (figure 2). Information about the epidemiology of HBV in Senegal was the main topic, emerging from 60 (38.2%) articles. 26.2% (40/157) articles focused on HBV-related health policies and the importance of strengthening the health system by increasing resources and funding for the National Hepatitis Control Programme. 17 articles (10.8%) focused on non-biomedical treatments and traditional medicine, most often emphasising recommendations on foods to avoid (ie, oil seeds) and diets to adopt (fruits and vegetables). News articles rarely focused on recommended biomedical treatments, accounting for only 1.9% of them. Only five articles (3.2%) featured interviews with healthcare experts, who identified key themes such as modes of transmission and the signs or complications associated with the disease. Vaccination was mentioned in 6.4% of the articles and emerged as the primary topic in discussion of the biomedical aspects of HBV. While biomedical experts highlighted the high cost of medical care, they did not specify any treatment recommendations or indicate where patients could access these treatments. Four articles included patients’ testimonials about their experiences with HBV, which were often reported in a fear-inducing style by the media.

The representatives of ‘Saafara Hépatites’, the only association of people living with hepatitis B in Senegal, were often primarily willing to testify publicly in the media. This included the former president, who demonstrated commitment and determination.

There is no communication about this disease. People don't know where to get tested, where to get vaccinated, none of it. It’s disgusting in a country like ours. *Ibrahima Gueye*

Ten articles featured information about the activities of The National Hepatitis Programme, emphasising its history, accomplishments and challenges. These articles predominantly included insights from an interview with the programmes’ director conducted during World Hepatitis Day.

### Readers’ comments features

The reactions left by readers often showed their quest for general information about HBV, including questions about treatments and requests for contact information for alternative practitioners. This virtual space evolved into a forum where commenters proposed various (unverified) treatments, often based on herbal remedies or spiritual practices.

Good evening dad, can a pregnant woman take the medication you suggest?

Cure hepatitis B and C on WhatsApp number 0022997XXXXXX all our products are made on the basis of plants and very effective without side effects.

A commonly observed practice was the inclusion of largely unverifiable testimonials by former patients, as well as patients’ names (possibly fictitious) in the comments, as a strategy for attracting and earning readers’ trust. Nonetheless, these practices were not exempt from denunciation and fraud accusations by other readers. Moreover, a virtual community emerged and displayed solidarity serving as a platform for mutual support, denouncing pseudo-healers, scammers and expressing condemnation towards the inadequate official communication. However, in the absence of health experts, these virtual discussion spaces did little to raise awareness about the disease.

“How can we avoid this disease since we are in a poor country where the state never discusses these kinds of diseases?” Anonymous commenter

Government institutions frequently became targets of intense criticism from readers, who turned these spaces into arenas of political defiance.

## Discussion

Our research revealed that the lack of accurate knowledge and representations of HBV, coupled with widespread misconceptions and rumours, is leading to a complex array of fears among people living with HBV in Senegal. This situation caused apprehension among participants, who faced uncertainties about the possibility of a cure, available treatment options, modes of transmission and the identification of specific signs or symptoms of the disease. In their search for reliable information, individuals often turned to the digital media, where they found unstructured content that amplified their fears. Furthermore, we found that online media content often provided a selective and narrow scope of HBV information, mainly focused on the epidemiology of the disease and the existing challenges in its management at a national level. However, these platforms also functioned as informal spaces for non-accredited health-sector actors to promote unverified treatments. The readers’ reaction spaces offered a platform for the circulation of new information, although without the assurance of its verification.

The absence of clear representations of HBV was closely related to the lack of formalised and structured health information sources, as well as the presence of conflicting oral narratives. Similar to findings from a recent study in Uganda, the absence of a vernacular term for HBV seemed to limit comprehension of the disease among lay populations, which may also have prevented stigma.^23^ In the limited existing literature from the West African context, representations of hepatitis B have not been directly associated with the disease itself, but rather with the complications of the end-stage disease. Notably, these symptoms often exhibit cross-pathological characteristics, appearing similarly across various medical conditions. This interpretation supports the premise that hepatitis B, as a distinct entity, remains largely obscure within the collective understanding.^18 19 24 25^ Moreover, the observable clinical signs linked to complications of HBV end-stage disease, which are shared with other diseases, contribute to the emergence of palpable fear, thereby introducing the potential for indirect social stigmatisation.^17 18^ The fear surrounding HBV transmission exhibited gendered differences, with married men feeling responsible for the ‘blood’ of their offspring and expressing concerns about the potential hereditary transmission of HBV. The men’s understanding of HBV blood transmission, within the framework of an ‘organic patriarchy’ refers to the social belief that men pass on their blood and diseases to their children through paternal genetic inheritance. Among women, a sense of vulnerability seemed to stem from the fear of transmitting the disease during pregnancy or breastfeeding.^26 27^

The traditional media (radio and TV), as well as informal knowledge networks, were presented as favoured sources of HBV information among participants, as similarly reported in several studies from Ghana.^28^,^30^ The content conveyed by these media was often perceived as threatening and accentuated the fear that official health communication strategies attempted to respond to.^28^ In line with previous studies from Senegal, Nigeria and Ghana, the internet emerged as the primary source of health information among young individuals. This increasing importance of digital platforms in the domain of public health information emphasises the need for their effective use to leverage health communication strategies.^1131^,^33^ Despite the existence of health-centred broadcasts for the general Senegalese population, official information is frequently overshadowed by self-promotional interventions from unaccredited practitioners offering unverified therapeutic services. In the Senegalese context, where resources allocated to health communication and awareness promotion are limited, individuals often experienced significant uncertainty regarding their condition.^11^ The diagnosis of hepatitis B triggered a cycle of fear and confusion among many individuals without adequate initial medical information, who frequently turned to the internet for guidance. In families with a known history of HBV, there is a disconnect between the potential for intergenerational knowledge transmission and the actual understanding of the infection, revealing a significant gap in awareness. While our data indicated that the awareness of a family history encouraged members to pursue screening, families were often overlooked as primary sources of information. Instead, online media emerged as the predominant channel for disseminating knowledge about the disease, leading some individuals to reach traditional healers or encounter concerning statistics about HBV prevalence in Senegal. This dynamic fostered an environment of uncertainty, trapping individuals in a vicious cycle of information-seeking, media consumption and heightened anxiety. Moreover, the unanswered concerns that emerged from interviewed individuals mirrored those asked by anonymous readers in the online comment spaces, underscoring the shared experiences and concerns among the general population and those living with HBV.

The high concentration of online media articles covering World Hepatitis Day and the prominence of the National Hepatitis Programme in news publications are consistent with the classic ‘agenda-setting’ mass media model, which proposes that the media play a crucial role in influencing the public agenda by selectively highlighting certain issues and events.^34 35^ Through the prioritisation of institutional health campaigns and public statements from health institution representatives, while neglecting other factors such as behaviour-focused messages or the promotion of preventive measures, the media in Senegal exert influence on the social context of the HBV epidemic. This influence, in turn, has the potential to shape public perceptions, individual behaviour and policy agendas.^36^ Moreover, for a comprehensive assessment of the impact on HBV media reporting, it is crucial to conduct a thorough examination of journalism norms and practices, as well as the diverse pressures imposed on the media by different stakeholders.^24^

In the context of mitigating misinformation, the readers’ comment spaces within news articles serve as a valuable platform for engagement, facilitating dialogue and the exchange of personal experiences. However, in the absence of effective moderation, these spaces become conducive environments for the propagation and dissemination of conflicting or contradictory messages. Although efforts and organisations aimed at combating misinformation in African contexts, such as Science Ebola and Africa check, have existed previously, it is the recent SARS-CoV-2 pandemic that has catalysed the establishment of transnational partnerships focused on addressing this issue.^37^,^39^ Despite this, virtual communities often serve as resilient ‘networks of support’ driven by a collective concern for public health matters.^40^ These communities actively caution against fraudulent intentions and guide readers towards reliable and credible sources of information.^41^ However, the influence of these factors on HBV communication patterns and individuals’ behaviours within the African context remains largely unexplored.

Our study presents novel data that contributes to the limited existing evidence on HBV knowledge and information access strategies among populations affected by HBV in West Africa. By employing a systematic approach that combined qualitative and quantitative data, we ensured a rigorous analysis and accurate interpretation of the collected information, providing valuable insights into the dynamics of information-seeking behaviours and the media landscape surrounding HBV. However, the absence of exploration into the experiences of other crucial stakeholders in health communication, such as digital media journalists and healthcare professionals limits the scope of our findings. Additionally, the inclusion of participants who had prior engagement with other healthcare services which may have influenced their experiences and knowledge potentially limited the generalisability of our findings to the broader HBV population. The instability of online media archives and the frequent republication of digital articles on Senegalese online media platforms may have limited the comprehensiveness of the content analysis. While thematic analysis of online content facilitates the identification and interpretation of patterns in textual data, it has significant shortcomings in capturing the full nuances of content. Moreover, our content analysis focused on primary themes but did not thoroughly explore the entire range of topics present in the online media articles. Finally, the reliance on Google for sourcing articles from Senegalese media may have introduced a systematic bias, restricting the retrieval of all pertinent publications. In an effort to minimise the potential for bias, we employed a dual, ‘forward’ and ‘backward’ approach. The systematic entering of selected keywords into the search interfaces of previously identified online media platforms to uncover additional articles that may have otherwise been overlooked.

## Conclusions

Significant knowledge gaps regarding HBV can be attributed to the lack of accurate representations, which results in a limited understanding of the condition among the people of Senegal. Consequently, the media often hinder the development of comprehensive knowledge, instilling fear and affecting individuals’ ability to actively engage in care for hepatitis B. It is crucial to prioritise the implementation of strategies and regulatory processes that enhance the quality of information within the media landscape and the broader public health discourse in Senegal.

## supplementary material

10.1136/bmjopen-2024-085453 online supplemental file 1

10.1136/bmjopen-2024-085453 online supplemental file 2

10.1136/bmjopen-2024-085453 online supplemental file 3

10.1136/bmjopen-2024-085453 online supplemental figure 1

## Data Availability

Data are available on reasonable request.
