# Supplementary material for: Understanding knowledge and media influence on people with hepatitis B in Senegal: a mixed-methods study
Source: BMJ Open. 2025 Mar 24;15(3):e085453. doi: 10.1136/bmjopen-2024-085453 (PMC11934370; doi:10.1136/bmjopen-2024-085453)
Supplement: online supplemental file 1 [file bmjopen-15-3-s001.docx]

**Semi-structured Interview Guide**

# Sociodemographic characteristics

| Date of the interview |  |
| --- | --- |
| Name, Surname |  |
| Age |  |
| Sex |  |
| Place of residence |  |
| Marital status |  |
| Number of children |  |
| Employment |  |
| Monthly income |  |
| Date of HBV diagnosis |  |

# SECTION A_**Background**

1. What comes to mind when you hear the term "hepatitis B"?
2. What questions do you have regarding your health condition?

# SECTION B_**Understanding of HBV and Healthcare-Seeking Behaviour**

1. What condition did you believe you had before learning about your positive status?
2. What was your initial treatment approach? Please share your experience.
3. Had you ever encountered the term hepatitis B prior to this?

# SECTION C_**HBV positive status announcement**

1. Please share how you became aware of your HBV infection, including details such as the year, location, and circumstances surrounding your diagnosis.
2. What prompted you to seek screening at that particular time, rather than earlier?
3. Who communicated your status to you, and what information did they provide? Please describe the terminology they used.
4. How do you feel about the way your status was announced to you?
5. What was your immediate reaction following the announcement?
6. What questions did you pose to the person who informed you of your status?
7. In your opinion, what are the barriers to screening individuals for HBV?
8. How do you evaluate the current state of hepatitis B screening? Please consider aspects such as cost, accessibility, and available facilities.
9. In your view, how could these barriers be addressed?

# SECTION D_**Disclosure**

1. Did you share your status after the announcement? If so, with whom, and what is your relationship with that person?
2. How did they react to your news?
3. What were your reasons for sharing your status? Did you share it partially or fully?
4. Did you encourage your close ones to get tested? If so, why?
5. Where did your family get tested, and what was the process? Did they get tested at the same time, one by one, or on the same day?
6. What have been your experiences with stigma related to the disease?

# SECTION E_**Attitudes, community life, and transmission risk management**

1. How do you believe you contracted this disease?
2. What are the internal and external manifestations of your condition?
3. Since learning of your diagnosis, have you altered your attitude towards your loved ones?
4. How do you manage the risks of transmission within your family?

- What precautions do you take regarding sexual activity?
- How has your diet changed in relation to your condition?
- What measures do you take when sharing utensils (such as spoons, glasses, etc.)?

1. Have you discussed hepatitis B with others? Please share your experience.
2. Do you have ancestors or relatives who have been affected by this condition? If so, please tell us about them.
3. What event related to your disease has had the most significant impact on you?

# SECTION F_**Treatments**

1. Now, let's discuss the treatments you have pursued since your diagnosis. I suggest we go through them one by one.
2. Where did you seek your first treatment?
3. How did you gather the resources to finance this treatment?
4. Who assisted you with this treatment?
5. Can you describe how the treatment went? (Please include details about its duration, the products used, and the method of administration.)
6. What is your evaluation of this approach?
7. Where did you go for your second treatment pathway?

- Ask the same questions as for the first approach.

1. Where did you go for your third therapeutic pathway?

- Ask the same questions as for the first approach.
- Continue if the person had other therapeutic approaches.

1. According to you, what are the constraints for accessing care after status discovery?
2. What do you think are the solutions to overcome these constraints?

# SECTION G_**Experiences, knowledge and perceptions of hepatitis B**

1. What term do you use in Wolof to refer to hepatitis B?
2. In your opinion, what does the 'B' in hepatitis B signify? (Please elaborate on hepatitis A, B, and C.)
3. What do you believe are the causes of hepatitis B? (Consider discussing diet and supernatural influences.)
4. What signs of the disease do you recognise? (Please discuss the significance of the absence of signs.)
5. In your view, how does the disease progress? (Explore possible complications.)
6. Who do you consider to be at risk for hepatitis B? (Take into account age, sex, lifestyle, and behaviours.)
7. How do you believe one can protect themselves from hepatitis B?
8. Would you classify this as an epidemic or a disease? (Consider the distinction between old versus new and cyclical versus permanent.)
9. What treatments do you think are available for HBV? (Discuss options in biomedicine, traditional medicine, and treatments from North America and China.)
10. Do you believe a person with hepatitis B can be cured? Why or how?
11. Where do you go to seek information about your questions regarding hepatitis B?

- What led you to choose this channel?
- What information is provided about hepatitis B here?
- What are your thoughts on it?
- How do you respond when you receive information?
- Please ask these same questions for each of the information channels mentioned.

1. What rumours have you heard about hepatitis B?
2. In your opinion, what should be explained to people with hepatitis like yourself?

# SECTION H_**Vaccination and Treatment of Hepatitis B**

1. Have you ever been vaccinated against hepatitis B? If so, when did you receive the vaccination, how many doses did you have, and where was it administered? If not, could you explain why?
2. What are your thoughts on vaccination against hepatitis B?
3. What constraints exist regarding vaccination for certain populations, and what do you believe is the reason for these constraints?
4. In your opinion, how can these constraints be addressed?
5. What are your current expectations regarding treatment for hepatitis B?
6. Why have you come to the SEN-B for follow-up now, given that you tested positive on [insert date]?
7. Doctors describe hepatitis B as a chronic disease, indicating that its treatment may last a lifetime. What is your perspective on this?

# SECTION I_**To Conclude**

1. We are at the end of our interview; would you like to add something?
