# Supplementary material for: Understanding knowledge and media influence on people with hepatitis B in Senegal: a mixed-methods study
Source: BMJ Open. 2025 Mar 24;15(3):e085453. doi: 10.1136/bmjopen-2024-085453 (PMC11934370; doi:10.1136/bmjopen-2024-085453)
Supplement: online supplemental file 2 [file bmjopen-15-3-s002.docx]

**Content Analysis Form**

| **Variable** | **Description** | **Code** |
| --- | --- | --- |
| **Article ID** | Website Date (6 digit)_article # (2 digit) | YYMMDD_01 |
| **Coder** | Initials of coder | XXX |
| **Data entry** | Initials of data entry | XXX |
| **Website domain** | Mark the short website domain | Free text |
| **URL** | Copy the full URL of the article | Free text |
| **Day of publication** | DD | 01-31 |
| **Month of publication** | MM | 01-12 |
| **Year of publication** | YYYY | 1900-2024 |
| **Time of publication** | TT:TT | 00:00-24:00 |
| **Title** | Quote the complete title of the article | Free text |
| **Subheading** | Provide any subtitles that appear on the article page | Free text  NA if None |
| **Type of media** | Indicate the type of online media | 1=companion website  2=web-aggregator  3=pure-player  4=blogpost  5=broadcast  99=other |
| **Section** | Mark the section that the article appears in | 0=missing  1=national/regional news  2=latest news  3=society  4=sports  5=health  6=business  7=politics  8=international  9=economy  99=other |
| **Photos** | Note if photos are included as captions to the article | 0=no  1=yes |
| **Population focus** | Indicate the populations referred by the article (several possible options) | 1=general population  2=children  3=women  4=key populations  5=people with HBV  6=healthcare worker  99=other |
| **Global Theme** | Analyze the actions within the narrative and identify the underlying topic. If uncertain between themes, consider paragraph length as a determining factor. | Free text |
| **Sub-themes** | Specify if other secondary themes are identified | Free text |
| **Number of views** |  | 0=non available  1=available (Precise the number) |
| **General reactions box** | Mark if a section including a general reactions box is included as part of the article | 0=non available  1=available with subscription  2=available without subscription |
