# Supplementary material for: Understanding knowledge and media influence on people with hepatitis B in Senegal: a mixed-methods study
Source: BMJ Open. 2025 Mar 24;15(3):e085453. doi: 10.1136/bmjopen-2024-085453 (PMC11934370; doi:10.1136/bmjopen-2024-085453)
Supplement: online supplemental file 3 [file bmjopen-15-3-s003.docx]

**Content Analysis**

|  | **Author** | **Title** | **Date** | **Global** | **Subtheme** | **Populations** | **Type Media** |
| --- | --- | --- | --- | --- | --- | --- | --- |
| #1 | dakarmatin.com | a la découverte de cette maladie qui touche 10% de notre population et qui tue plus que le sida | 2018-07-29 | Prevalence |  | General population | Companion website |
| #2 | enqueteplus.com | dr abdoul aziz kasse, cancerologue : ‘’comment diminuer, voire éradiquer certains cancers les plus fréquents’’ | 2020-05-31 | Risk factors and complications | Prevention | General population | Companion website |
| #3 | enqueteplus.com | absence de vaccination contre les hépatites : 20% des sénégalais pourraient être atteints | 2013-07-22 | Prevention | Vaccination | General population | Companion website |
| #4 | enqueteplus.com | prise en charge des hépatites : aristide le dantec doté d’un nouvel appareil | 2016-12-24 | Policy | Laboratory capacities | General population | Companion website |
| #5 | enqueteplus.com | journee mondiale de lutte contre les hepatites : les acteurs plaident la gratuité du dépistage | 2018-07-30 | Screening | Treatment | General population | Companion website |
| #6 | enqueteplus.com | temoignage ibrahima gueye, president de l’association saafara hepatite : ‘’la première ordonnance qu’on m’a prescrite coûte 7,300 millions’’ | 2015-07-28 | Civil society advocacy |  | pwHBV | Companion website |
| #7 | enqueteplus.com | lutte contre les hepatites au senegal : la vérité sur le pnlh, un programme pas comme les autres son histoire | 2018-08-14 | Policy |  | General population | Companion website |
| #8 | enqueteplus.com | interview : professeur aminata sall diallo, coordinatrice du programme national de lutte contre les hepatites au senegal : ‘’nous n’avons pas du tout de budget pour la communication’’ | 2015-07-28 | Policy | Communication | General population | Companion website |
| #9 | enqueteplus.com | plus de 2 millions de senegalais exposes aux virus des hepatites : des campagnes de dépistage sont prévues | 2015-07-29 | Prevalence |  | General population | Companion website |
| #10 | enqueteplus.com | prevalence des infections virales au senegal : les hépatites, des pathologies négligées par les autorités | 2015-07-28 | Prevalence | Neglected | General population | Companion website |
| #11 | enqueteplus.com | lutte contre l’hepatite : le dépistage des femmes enceintes exhorté | 2020-07-30 | Screening |  | Women | Companion website |
| #12 | enqueteplus.com | pr souleymane mboup sur les hepatites virales : ‘’la vaccination a permis d&#039;éviter plus de 800 000 nouvelles infections chroniques’’ | 2016-01-20 | Prevention |  | Children | Companion website |
| #13 | enqueteplus.com | lutte contres les hepatites au sénégal : plus de 65 mille enfants vaccinés à la naissance | 2016-07-29 | Prevention | Children | Children | Companion website |
| #14 | enqueteplus.com | hépatite, journée mondiale contre l’hépatite, | 2020-07-28 | Prevalence |  | General population | Companion website |
| #15 | enqueteplus.com | journée mondiale de lutte contre les hépatites : la fondation sonatel sensibilise sur l&#039;affection | 2013-07-29 | Civil society advocacy | Stakeholders | pwHBV | Companion website |
| #16 | ferloo.com | au congrès mondial sur le sida, les hépatites veulent gagner en visibilité | 2017-07-24 | Policy | Conference | Key populations | Companion website |
| #17 | ferloo.com | hépatites : "2 millions de sénégalais sont exposés", selon le directeur de la santé | 2015-07-27 | Prevalence |  | General population | Companion website |
| #18 | ferloo.com | « ce n’est pas sans raison qu’on appelle l’hépatite virale le «tueur silencieux» | 2015-09-12 | Prevalence | Policy | General population | Companion website |
| #19 | ferloo.com | l’oms recommande l’adoption de mesures d’urgence contre l’hépatite | 2015-07-27 | Policy | Efforts | General population | Companion website |
| #20 | igfm.sn | "1 500 000 sénégalais sont des porteurs chroniques de l'hépatite b" | 2019-07-28 | Prevalence |  | General population | Companion website |
| #21 | lequotidien.sn | pr abdoul aziz kassé tire la sonnette d’alarme : «le cancer est la première cause de décès après l’âge de 40 ans au sénégal» \| lequotidien journal d'informations générales | 2019-12-11 | Risk factors and complications | Alert | General population | Companion website |
| #22 | lequotidien.sn | santé - augmentation du nombre de cas et des décès : les ravages du cancer | 2022-02-10 | Risk factors and complications | Mortality | General population | Companion website |
| #23 | lequotidien.sn | congrès mondial sur le sida : les hépatites veulent gagner en visibilité | 2017-07-29 | Policy |  | Key populations | Companion website |
| #24 | lequotidien.sn | lutte contre l’hépatite b : le traitement subventionné à 5 000 francs cfa | 2017-07-29 | Policy | PNLH | pwHBV | Companion website |
| #25 | lequotidien.sn | campagne de lutte contre les hépatites : les autorités médicales ciblent 1325 agents de santé à dépister | 2019-07-26 | Screening | Health workers | Health care workers | Companion website |
| #26 | lequotidien.sn | révélations de aminata sall diallo, coordinatrice du pnlh : «près de deux millions de porteurs des virus de l’hépatite b et c» | 2018-07-23 | Prevalence | PNLH | General population | Companion website |
| #27 | lequotidien.sn | lancement de la nouvelle carte de pointage contre l’hépatite virale : l’oms décèle des faiblesses dans la réponse à la lutte | 2019-06-18 | Policy |  | General population | Companion website |
| #28 | lesoleil.sn | carrière scientifique : pr. aminata sall diallo, un modèle pour les filles | 2019-07-18 | Policy | PNLH | General population | Companion website |
| #29 | lesoleil.sn | lutte contre les hépatites : la prévalence baisse de 65 % à 3 % chez les enfants | 2019-07-29 | Prevalence | Children | General population | Companion website |
| #30 | lesoleil.sn | région de kaffrine : 13 % des agents de santé positifs à l’hépatite b | 2020-02-06 | Prevalence |  | Health care workers | Companion website |
| #31 | lesoleil.sn | douleurs aux côtes, jaunissement des muqueuses, des urines et courbatures… : ces signes des hépatites qui doivent alerter | 2021-11-08 | Risk factors and complications |  | pwHBV | Companion website |
| #32 | sudonline.sn | l’hepatite en afrique une maladie de sante publique | 2016-01-20 | Policy | Policy | General population | Companion website |
| #33 | tambacounda.info | les hépatites gagnent du terrain au sénégal | 2018-07-27 | Prevalence |  | General population | Companion website |
| #34 | tambacounda.info | tambacounda : introduction du vaccin contre l’hépatite b dans le pev, la maladie tue prés d’un million de personnes par an. | 2016-02-23 | Prevention |  | Children | Companion website |
| #35 | actunet.net | quel régime alimentaire contre les hépatites | 2018-04-23 | Traditional medicine |  | General population | Pure-player |
| #36 | actunet.net | pénurie de vaccins contre l’hépatite b | 2017-03-03 | Prevention | Vaccin | Children | Pure-player |
| #37 | africacheck.org | non, le bois dartre ne guérit pas l’hépatite b en trois jours | 2019-12-09 | Policy |  | General population | Pure-player |
| #38 | africacheck.org | aucune preuve que le miel, la cannelle et la gelée royale guérissent les hépatites b et c | 2019-09-19 | Policy |  | pwHBV | Pure-player |
| #39 | banlieueweb.com | 87% des sénégalais ont un contact avec le virus de l'hépatite | 2019-07-27 | Prevalence | Symptoms | General population | Pure-player |
| #40 | dakar7.com | hépatites ; deux millions de personnes exposées au sénégal | 2015-07-28 | Prevalence |  | General population | Pure-player |
| #41 | dakar7.com | hépatite b et c : l'etat inquiet du taux de prévalence de cette maladie | 2019-07-04 | Prevalence |  | General population | Pure-Player |
| #42 | dakar7.com | hépatite b: 2 millions de cas au sénégal | 2017-07-28 | Prevalence |  | General population | Pure-player |
| #43 | dakar7.com | hépatites ; deux millions de personnes exposées au sénégal | 2015-07-28 | Prevalence |  | General population | Pure-player |
| #44 | dakar7.com | hépatites virales : des moyens de transmission souvent méconnus | 2016-07-11 | Risk factors and complications | Transmission | General population | Pure-player |
| #45 | dakaractu.com | pr moussa seydi : « au sénégal, une étude scientifique est en cours sur l'artémisia » | 2020-05-03 | Traditional medicine | Treatment | pwHBV | Pure-Player |
| #46 | dakaractu.com | hépatite: une maladie soignable selon le tradipraticien cheikh omar diop | 2016-01-31 | Traditional medicine | Treatment | pwHBV | Pure-Player |
| #47 | dakaractu.com | lutte contre l’hépatite : beaucoup d'efforts consentis par l'etat (awa marie coll seck) | 2017-07-28 | Policy | Efforts | General population | Pure-Player |
| #48 | dakarmidi.net | découvrez les conseils du tradipraticien abdoulaye ndiaye pour le traitement de l'hépatite b - | 2019-04-14 | Traditional medicine | Treatment | pwHBV | Pure-player |
| #49 | dakarprivee.com | kaffrine :un taux de prévalence de l' hépatite b fait froid sur le dos | 2019-11-27 | Prevalence |  | General population | Pure-player |
| #50 | dakarden.sn | prise en charge des hépatites virales b et c: le sénégal ne profite pas des opportunités offertes | 2015-07-28 | Policy | Opportunities | General population | Pure-player |
| #51 | echos-senegal.com | sénégal-hépatite b : le taux de prévalence estimé à 17% | 2015-07-30 | Prevalence |  | General population | Pure-player |
| #52 | fondationsonatel.com | la fondation sonatel accompagne la journée mondiale de lutte contre les hépatites | 2020-07-28 | Policy | Stakeholders | pwHBV | Pure-player |
| #53 | intelligences.info | pr souleymane mboup : «i'hépatite b est un silent killer...au sénégal, il y aura 50 000 décès entre 2016 et 2030 | 2017-08-14 | Risk factors and complications |  | pwHBV | Pure-player |
| #54 | intelligences.info | hépatites : pourquoi il y a alerte au sénégal | 2018-07-23 | Prevalence | Alert | General population | Pure-player |
| #55 | lactuacho.com | dommages causés par les maladies virales : les complications de l’hépatite b tuent plus que le sida – | 2018-07-28 | Risk factors and complications |  | pwHBV | Pure-player |
| #56 | lejecos.com | 80% des personnes vivant avec l’hépatite n’ont pas accès aux soins nécessaires, alerte l’oms | 2019-07-29 | Policy | Access to care | pwHBV | Pure-player |
| #57 | lejecos.com | sénégal: hépatite b - une urgence silencieuse | 2014-05-12 | Prevalence | Neglected | General population | Pure-player |
| #58 | leral.net | les plus grands spécialistes de l'hépatite se réunissent à dakar, au sénégal, lors du sommet africain sur l'hépatite virale | 2016-01-20 | Policy | Conference | pwHBV | Pure-player |
| #59 | leral.net | hépatite au sénégal: le nombre de malades estimé à...2,5 millions ! | 2019-12-03 | Prevalence | Alert | General population | Pure-player |
| #60 | leral.net | hépatite b: 10% de la population sénégalaise infectés | 2021-10-20 | Prevalence | Alert | General population | Pure-player |
| #61 | leral.net | baisse drastique du taux d’hépatite chronique au sénégal. | 2013-07-20 | Prevalence | Outcomes | General population | Pure-player |
| #62 | leral.net | journée mondiale: les hépatites gagnent du terrain au sénégal | 2018-07-27 | Prevalence | Alert | General population | Pure-player |
| #63 | leral.net | hépatite: l'oms exhorte les pays à investir dans la lutte | 2019-07-28 | Policy | Priority | General population | Pure-player |
| #64 | leral.net | traitement des hépatites virales : essayez le terminalia catappa, le tinospora, le tamarindus… | 2012-05-21 | Traditional medicine | Treatment | pwHBV | Pure-player |
| #65 | metrodakar.net | hépatite b: "10 % de la population sénégalaise sont des porteurs chroniques du virus" | 2018-07-27 | Prevalence |  | General population | Pure-player |
| #66 | metrodakar.net | dites adieu à l'hépatite b avec ça ! | 2018-03-05 | Traditional medicine | Treatment | pwHBV | Pure-player |
| #67 | nettali.com | hepatite b : plus de 2 millions de sénégalais porteurs du virus | 2019-07-27 | Prevalence |  | General population | Pure-player |
| #68 | pressafrik.com | dakar abrite la conférence internationale francophone "vih, hépatites et santé sexuelle, covid-19" (cnls) | 2020-11-05 | Policy | Conference | Key populations | Pure-player |
| #69 | pressafrik.com | santé : 2 millions de sénégalais vivent avec les hépatites. | 2015-07-28 | Prevalence |  | General population | Pure-player |
| #70 | pressafrik.com | recrudescence de l’hépatite b à touba : 1750 cas découverts sur 5000 consultés | 2016-08-21 | Prevalence | Blood bank | General population | Pure-player |
| #71 | pressafrik.com | la maladie des hépatites est "une sur-priorité au sénégal" | 2011-07-25 | Policy | Priority | General population | Pure-player |
| #72 | pressafrik.com | ​journée mondiale de l’hépatite : 2 millions de sénégalais sont porteurs de cette maladie | 2018-07-29 | Prevalence |  | General population | Pure-player |
| #73 | reussirbusiness.com | lutte contre les hépatites : la fondation sonatel se mobilise | 2018-07-30 | Policy | Founders | General population | Pure-player |
| #74 | sante-senegal.com | hépatite b une maladie très contagieuse qui attaque le foie. | 2018-06-18 | Risk factors and complications | Risks | pwHBV | Pure-player |
| #75 | senegal7.com | hépatite b : le professeur aminata sall diallo fait le diagnostic - senegal7 | 2019-07-30 | Risk factors and complications | PNLH | pwHBV | Pure-player |
| #76 | senegal7.com | kaffrine : l’hépatite b fait des ravages - senegal7 | 2019-11-23 | Prevalence | Neglected | General population | Pure-player |
| #77 | senegal7.com | hépatite b : plus de 2 millions de sénégalais porteurs du virus - senegal7 | 2019-07-27 | Prevalence | Risk factors | General population | Pure-player |
| #78 | senegal7.com | santé- docteur, mamadou guèye : « un sénégalais sur dix est infecté par l’hépatite b » - senegal7 | 2018-07-30 | Prevalence | Prevalence | General population | Pure-player |
| #79 | senegalblackrainbow.org | ce mal méconnu: l’hépatite | 2018-07-28 | Prevalence | Information | General population | Pure-player |
| #80 | senegaldirect.com | la plante qui traite le cancer, l’hépatite, les maladies du foie, les reins, les estomes, voici comment préparer | 2019-11-20 | Traditional medicine | Treatment | pwHBV | Pure-player |
| #81 | senenews.com | près de deux millions d’individus porteurs des virus de l’hépatite b et c au sénégal | 2018-07-21 | Prevalence |  | General population | Pure-player |
| #82 | senenews.com | hépatite b : 10% de la population sénégalaise infectés | 2012-08-01 | Prevalence | Blood bank | General population | Pure-player |
| #83 | senenews.com | diabète, hépatite b, cholestérol : séances gratuites de dépistage au cices, à partir de mardi | 2019-12-16 | Screening |  | General population | Pure-player |
| #84 | senenews.com | awa marie colle seck introduit le vaccin contre l'hépatite b dans le programme élargi de la vaccination nationale | 2016-02-20 | Prevention | Children | Children | Pure-player |
| #85 | senenews.com | l’hépatite b entraîne plus de dommages que l’infection à vih | 2018-07-29 | Civil society advocacy | Outcomes | pwHBV | Pure-player |
| #86 | seneplus.com | les hépatites, des pathologies négligées par les autorités | 2015-07-28 | Prevalence | Neglected | General population | Pure-player |
| #87 | seneplus.com | la prevalence des porteurs de l’hepatite estimee a 11% au senegal | 2015-07-28 | Prevalence |  | General population | Pure-player |
| #88 | seneplus.com | l’introduction du vaccin contre l’hepatite b a la naissance effective | 2016-02-03 | Prevention |  | Children | Pure-player |
| #89 | setal.net | don : la fondation servir le sénégal reçoit 16 .000 doses d’immunoglobuline contre l’hépatite b | 2013-08-07 | Treatment and care | Stakeholder | pwHBV | Pure-player |
| #90 | sneipsofficiel.net | kenkelibaa/ santé hépatite b (sneips) | 2016-06-31 | Traditional medicine | Treatment | pwHBV | Pure-player |
| #91 | teranganews.sn | hépatite b, un virus répandu chez les jeunes mais méconnu | 2017-12-08 | Prevalence | Young | General population | Pure-player |
| #92 | thieydakar.net | co-infection vih et hepatites : le cocktail de tous les dangers | 2019-11-26 | Policy |  | Key populations | Pure-player |
| #93 | thieydakar.net | sida et hépatites : les défis qui interpellent les acteurs | 2019-07-02 | Policy |  | Key populations | Pure-player |
| #94 | thieydakar.net | sida et hépatites : les 13e journées scientifiques prévues ce mardi | 2019-06-27 | Policy |  | Key populations | Pure-player |
| #95 | thieydakar.net | hepatite : 4,5 millions de décès d’ici à 2030 | 2019-07-27 | Risk factors and complications |  | pwHBV | Pure-player |
| #96 | thieydakar.net | controverses autour de la lutte contre les hépatites - | 2018-08-17 | Policy | Neglected | General population | Pure-player |
| #97 | thieydakar.net | lutte contre les hépatites: le pnlh, un programme pas comme les autres | 2018-08-13 | Policy | Neglected | General population | Pure-player |
| #98 | thieydakar.net | hépatites : pr aminata sall diallo annonce la décentralisation de la prise en charge | 2018-07-21 | Policy | PNLH | pwHBV | Pure-player |
| #99 | thieydakar.net | taux de prévalence élevé de l'hépatite b à kaffrine | 2019-11-23 | Prevalence |  | General population | Pure-player |
| #100 | thieydakar.net | hepatite : 325 millions de personnes infectées dans le monde | 2019-07-27 | Prevalence |  | General population | Pure-player |
| #101 | thieydakar.net | hépatite b et diabète: la fidak veut dépister 600 personnes | 2017-12-27 | Screening |  | General population | Pure-player |
| #102 | thieydakar.net | l'hépatite, une inflammation du foie aux multiples visages | 2019-07-29 | Risk factors and complications |  | pwHBV | Pure-player |
| #103 | thieydakar.net | santé : 323 millions de personnes dans le monde entier souffrent de l'hépatite (oms) | 2018-07-29 | Prevalence |  | General population | Pure-player |
| #104 | thieydakar.net | la journée mondiale contre l'hépatite célébrée à niakhar (fatick) | 2018-07-28 | Policy |  | General population | Pure-player |
| #105 | wathi.org | «beaucoup de jeunes se promènent avec l’hépatite b sans le savoir au sénégal» | 2017-06-09 | Prevention |  | pwHBV | Pure-player |
| #106 | xibaaru.sn | hepatite b : plus de 2 millions de sénégalais porteurs du virus | 2019-08-22 | Prevalence | Transmission | General population | Pure-player |
| #107 | actusen.sn | elimination du vih et des hépatites : le dr ibra ndoye donne les pistes à suivre | 2019-07-02 | Policy |  | General population | Web-aggregator |
| #108 | actusen.sn | santé: l’oms appelle à investir dans l’élimination de l’hépatite | 2019-07-27 | Policy |  | General population | Web-aggregator |
| #109 | actusen.sn | 745 patients consultés, 26 cas de diabète décelés et 5 personnes atteintes de l’hépatite b identifiées | 2016-04-12 | Prevalence |  | General population | Web-aggregator |
| #110 | aDakar.com | environ 2 millions de personnes sous la menace des hépatites au sénégal | 2015-07-27 | Prevalence |  | General population | Web-aggregator |
| #111 | aDakar.com | deux millions de sénégalais exposés aux hépatites (officiel) | 2015-07-28 | Risk factors and complications | Transmission | General population | Web-aggregator |
| #112 | aDakar.com | environ 2 millions de personnes sous la menace des hépatites au sénégal | 2015-07-27 | Risk factors and complications | Transmission | General population | Web-aggregator |
| #113 | allafrica.fr | sénégal: conférence internationale sur les hépatites, mardi à dakar | 2011-07-25 | Policy |  | General population | Web-aggregator |
| #114 | allafrica.fr | sénégal: le directeur de la santé conseille le dépistage général et ciblé de l'hépatite | 2012-07-30 | Screening | Screening | General population | Web-aggregator |
| #115 | allafrica.fr | sénégal: hepatite b - un malade témoigne sur sa vie d'«enfer» | 2013-07-30 | Civil society advocacy |  | pwHBV | Web-aggregator |
| #116 | allafrica.fr | sénégal: les hépatites virales - une avancée considérable dans leur traitement par les plantes ! | 2009-09-05 | Traditional medicine | Treatment | pwHBV | Web-aggregator |
| #117 | journaldusenegal.com | afrique : 300 personnes décèdent chaque jour des complications liées à l'hépatite b et c (responsable) | 2020-07-28 | Prevalence |  | General population | Web-aggregator |
| #118 | kewoulo.info | santé : l'hépatite b crée plus de dommages que le vih | 2018-07-30 | Prevalence | Prevalence | General population | Web-aggregator |
| #119 | koldanews.com | le cancer du foie du à l'hépatite b entraînent plus de dommages que le sida au sénégal | 2018-07-29 | Risk factors and complications |  | pwHBV | Web-aggregator |
| #120 | koldanews.com | serigne modou gueye gandigal nous parle de l'hépatite b: causes et remèdes | 2020-05-04 | Traditional medicine | Treatment | pwHBV | Web-aggregator |
| #121 | koldanews.com | près d’1,5 million de sénégalais souffriraient de l'hépatite b | 2019-07-29 | Prevalence |  | General population | Web-aggregator |
| #122 | koldanews.com | deux millions de sénégalais porteurs des virus des hépatites b et c | 2018-07-23 | Prevalence |  | General population | Web-aggregator |
| #123 | laviesenegalaise.com | deux millions de personnes sont porteuses chronique de l'hépatite au sénégal | 2017-07-28 | Prevalence |  | General population | Web-aggregator |
| #124 | laviesenegalaise.com | santé : les chiffres alarmants de l'hépatite b | 2019-07-28 | Prevalence | Alert | General population | Web-aggregator |
| #125 | laviesenegalaise.com | touba : recrudescence de l’hépatite b, 1750 cas découverts sur 5000 consultés | 2016-08-21 | Prevalence | Alert | General population | Web-aggregator |
| #126 | lejournaldelafrique.com | au sénégal, l’hépatite b à l’épreuve des fake-news | 2022-01-28 | Policy |  | General population | Web-aggregator |
| #127 | ndarinfo.com | aminata sall diallo, professeur agrége en physiologie: "je suis très fière d’être née à saint-louis" | 2015-09-10 | Policy | PNLH | General population | Web-aggregator |
| #128 | ndarinfo.com | ​journée mondiale de l’hépatite : 2 millions de sénégalais sont porteurs de cette maladie | 2019-07-29 | Policy | Prevalence | pwHBV | Web-aggregator |
| #129 | sanslimitesn.com | deux millions de sénégalais sont porteurs des virus... | 2018-07-21 | Prevalence |  | General population | Web-aggregator |
| #130 | sanslimitesn.com | dites adieu à l’hépatite b avec ça ! | 2018-03-05 | Traditional medicine | Treatment | pwHBV | Web-aggregator |
| #131 | sen360.sn | hépatite b au sénégal: 10% de la population infectée | 2021-10-20 | Prevalence |  | General population | Web-aggregator |
| #132 | sen360.sn | hépatite b et c: chercheurs, ong et pays africains tirent la sonnette d'alarme | 2018-04-04 | Policy | Priority | General population | Web-aggregator |
| #133 | sen360.sn | santé : la situation des personnes malades d’hépatite avec ibrahima gueye pdt asso. safara hépatite – 2stv | 2019-07-27 | Civil society advocacy | Prevalence | pwHBV | Web-aggregator |
| #134 | senego.com | l'afrique compte 100 millions de porteurs des virus de l'hépatite virale (spécialistes) | 2016-01-19 | Prevalence |  | General population | Web-aggregator |
| #135 | senego.com | hépatite b : 2,5 millions de personnes souffrent de cette maladie au sénégal | 2015-09-07 | Prevalence |  | General population | Web-aggregator |
| #136 | senego.com | santé - touba: sur 5000 donneurs de sang, 1750 sont affectées par l'hépatite b | 2016-08-21 | Prevalence | Blood bank | General population | Web-aggregator |
| #137 | senetoile.net | prise en charge de l’hépatite b et c le sénégal peaufine sa stratégie | 2015-07-29 | Policy |  | pwHBV | Web-aggregator |
| #138 | senetoile.net | santé-rupture du ténofovire à dakar | 2014-09-27 | Treatment and care | Stock out | pwHBV | Web-aggregator |
| #139 | seneweb.com | hépatite b au sénégal: 10% de la population infectée | 2021-10-20 | Prevalence | Senegal | General population | Web-aggregator |
| #140 | seneweb.com | près de deux millions de porteurs de virus de l’hépatite b et c | 2018-07-21 | Prevalence | PNLH | General population | Web-aggregator |
| #141 | seneweb.com | lutte contre l'hépatite b : les 17 % de porteurs chroniques invités à faire un bilan tous les 6 mois | 2013-03-12 | Policy | Prevention | pwHBV | Web-aggregator |
| #142 | seneweb.com | hépatite b – une urgence silencieuse au sénégal | 2014-05-13 | Policy | Priority | General population | Web-aggregator |
| #143 | seneweb.com | hépatite b et c : prudence au percing, tatouage, à l’excision, à la circoncision, aux matériels de soins à usage unique… | 2011-07-23 | Risk factors and complications | Transmission | General population | Web-aggregator |
| #144 | seneweb.com | la maladie de l'hépatite : causes et traitements b part1 | 2012-08-28 | Treatment and care | Causes | pwHBV | Web-aggregator |
| #145 | seneweb.com | introduction du vaccin contre l’hépatite b dans le programme élargi de vaccination | 2016-02-20 | Prevention |  | Children | Web-aggregator |
| #146 | seneweb.com | hépatite b. des patients alertent sur une « pénurie » de vaccins | 2017-02-15 | Prevention | Stock out | Children | Web-aggregator |
| #147 | seneweb.com | Journée mondiale de lutte contre hépatite. | 2018-07-15 | Policy |  | General population | Web-aggregator |
| #148 | seneweb.com | absence de la dose de naissance du vaccin anti-hépatite b : cette menace qui se profile à l’horizon après la covid-19 | 2020-07-27 | Prevention | Stock out | Children | Web-aggregator |
| #149 | senxibar.com | ibrahima guèye, président de l’association saafara hépatite: "la première ordonnance qu’on m’a prescrite coûte 7,300 millions" | 2015-07-29 | Civil society advocacy |  | pwHBV | Web-aggregator |
| #150 | teranganews.sn | fatick-prévention : quelques 1325 agents de santé ciblés par une campagne de dépistage contre l’hépatite | 2019-07-26 | Screening | Prevalence | Health care workers | Web-aggregator |
| #151 | xalima.com | virus de l'hépatite b. plus de deux millions de sénégalais atteints | 2010-05-18 | Prevalence |  | General population | Web-aggregator |
| #152 | xibar.net | hépatite b : 17 % des sénégalais sont atteints d’une hépatite b chronique | 2019-05-19 | Prevalence |  | General population | Web-aggregator |
| #153 | leblocafrique.com | désormais possible de mesurer la charge virale de l'hépatite b au sénégal au prix de 15000fcfa | 2016-12-24 | Policy |  | General population | Pure-player |
| #154 | leuzetv.com | conseil santé: l'hépatite b une maladie qu'on peut éviter grace à . | 2018-06-14 | Traditional medicine | Treatment | General population | Pure-player |
| #155 | psej.sn | lutte contre les hépatites: une priorité nationale de santé publique au sénégal | 2018-09-27 | Policy |  | General population | Pure-player |
| #156 | rts.sn | hepatite : interview pr. aminata sall diallo | 2012-08-01 | Prevalence | Policy | General population | Pure-player |
| #157 | yesdakar.com | l'hépatite une maladie bien présent au senegal. quelles solutions selon ibrahima gueye | 2019-08-10 | Civil society advocacy | Prevalence | pwHBV | Pure-player |
