# Supplementary figures and images for: Understanding knowledge and media influence on people with hepatitis B in Senegal: a mixed-methods study
Source: BMJ Open. 2025 Mar 24;15(3):e085453. doi: 10.1136/bmjopen-2024-085453 (PMC11934370; doi:10.1136/bmjopen-2024-085453)

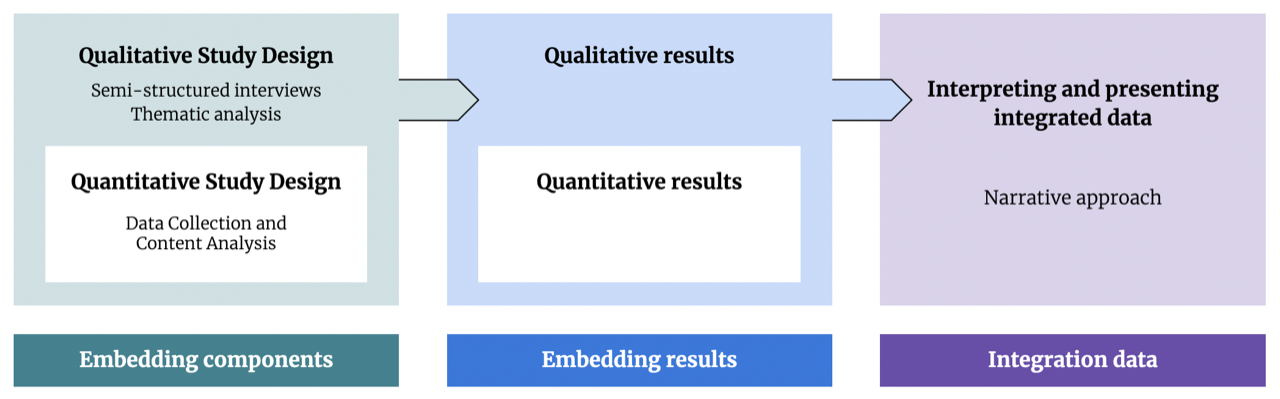

Supplement: online supplemental figure 1 [file bmjopen-15-3-s004.png]
